# A new transcriptome and transcriptome profiling of adult and larval tissue in the box jellyfish *Alatina alata*: an emerging model for studying venom, vision and sex

**DOI:** 10.1186/s12864-016-2944-3

**Published:** 2016-08-17

**Authors:** Cheryl Lewis Ames, Joseph F. Ryan, Alexandra E. Bely, Paulyn Cartwright, Allen G. Collins

**Affiliations:** 1 Department of Invertebrate Zoology, National Museum of Natural History, Smithsonian Institution, Washington, DC 20013 USA; 2 Biological Sciences Graduate Program, University of Maryland, College Park, MD 20742 USA; 3 Whitney Laboratory for Marine Bioscience, University of Florida, St Augustine, FL 32080 USA; 4 Department of Biology, University of Florida, Gainesville, FL 32611 USA; 5 Department of Biology, University of Maryland, College Park, MD USA; 6 Department of Ecology and Evolutionary Biology, University of Kansas, Lawrence, KS 66045 USA; 7 National Systematics Laboratory, NOAA Fisheries, National Museum of Natural History, Smithsonian Institution, Washington, DC USA

**Keywords:** Cubozoa, Expression patterns, Pedalium, Sting, Embryo, Gametogenesis, Planulae, Eye, Spawning aggregations, Sperm

## Abstract

**Background:**

Cubozoans (box jellyfish) are cnidarians that have evolved a number of distinguishing features. Many cubozoans have a particularly potent sting, effected by stinging structures called nematocysts; cubozoans have well-developed light sensation, possessing both image-forming lens eyes and light-sensitive eye spots; and some cubozoans have complex mating behaviors, including aggregations, copulation and internal fertilization. The cubozoan *Alatina alata* is emerging as a cnidarian model because it forms predictable monthly nearshore breeding aggregations in tropical to subtropical waters worldwide, making both adult and larval material reliably accessible. To develop resources for *A. alata*, this study generated a functionally annotated transcriptome of adult and larval tissue, applying preliminary differential expression analyses to identify candidate genes involved in nematogenesis and venom production, vision and extraocular sensory perception, and sexual reproduction, which for brevity we refer to as “venom”, “vision” and “sex”.

**Results:**

We assembled a transcriptome *de novo* from RNA-Seq data pooled from multiple body parts (gastric cirri, ovaries, tentacle (with pedalium base) and rhopalium) of an adult female *A. alata* medusa and larval planulae. Our transcriptome comprises ~32 K transcripts, after filtering, and provides a basis for analyzing patterns of gene expression in adult and larval box jellyfish tissues. Furthermore, we annotated a large set of candidate genes putatively involved in venom, vision and sex, providing an initial molecular characterization of these complex features in cubozoans. Expression profiles and gene tree reconstruction provided a number of preliminary insights into the putative sites of nematogenesis and venom production, regions of phototransduction activity and fertilization dynamics in *A. alata*.

**Conclusions:**

Our *Alatina alata* transcriptome significantly adds to the genomic resources for this emerging cubozoan model. This study provides the first annotated transcriptome from multiple tissues of a cubozoan focusing on both the adult and larvae. Our approach of using multiple body parts and life stages to generate this transcriptome effectively identified a broad range of candidate genes for the further study of coordinated processes associated with venom, vision and sex. This new genomic resource and the candidate gene dataset are valuable for further investigating the evolution of distinctive features of cubozoans, and of cnidarians more broadly.

**Electronic supplementary material:**

The online version of this article (doi:10.1186/s12864-016-2944-3) contains supplementary material, which is available to authorized users.

## Background

Cubozoa (box jellyfish) is a class of Cnidaria with a suite of distinct features including a cuboid bell, lens eyes and a typically highly potent sting. Like many cnidarians, cubozoan life history includes a swimming planula larva that ultimately settles onto a substrate to become an asexually reproducing polyp. Polyps then give rise to medusae (jellyfish), which have separate sexes and are the sexually reproductive stage. Some cubozoan taxa have evolved complex sexual behavior including synchronous spawning aggregations, mating and internal fertilization [1–3]. Cubozoan medusae vary widely in the potency of their sting; in humans, cubozoan stings range from being harmless to causing deadly envenomation [4–7]. A particularly note-worthy character of cubozoan medusae is their image-forming lens eyes, which have been implicated in visually-guided behavior [8–11].

Like all other cnidarians, cubozoans possess nematocysts (stinging organelles) essential for prey capture and defense. Nematocysts are remarkably complex subcellular structures that develop within specialized cells called nematocytes. Nematocysts are secreted from post-Golgi vesicles and consist of a double-walled capsule containing venom and a harpoon-like spiny tubule; and one to several different kinds can develop within a cnidarian throughout its life cycle [12]. Nematocysts are of several forms, broadly divided into penetrant (e.g., euryteles) and adherent (e.g., isorhizas). Penetrant nematocysts are primarily concentrated in the tentacles of cubozoan medusae where they are used for prey capture. In some species, nematocysts are also found in body parts with putative digestive roles, such as the gastric cirri (in the stomach), where they may further immobilize prey items inserted into the cubozoan mouth (manubrium) [13]. Adherent nematocysts are typically found on the exterior of the cubed-shaped bell and do not appear to function in predation there [14, 15]. The location of nematocyst development (nematogenesis) is poorly known in most cnidarians; having only been well-characterized in the model hydrozoan polyp *Hydra*, where morphology and molecular studies reveal clusters of developing nematocysts within the body [16]. In contrast, molecular studies of another hydrozoan medusa *Clytia,* suggest that nematogenic regions are found in the tentacle bulb, proximal to the tentacles in which mature nematocysts are found [17]. Transcriptomic and proteomic studies on the cubozoan *Chironex fleckeri*, the scyphozoans *Chrysaora fuscescens* and *Stomolophus meleagris*, and the hydrozoan *Olindias sambaquiensis* have focused on characterizing venom components from tentacle components [5, 18–21], but it is unknown whether nematogenesis and venom production occur solely in the medusa tentacles. In *A. alata*, tiny unidentified nematocysts have been documented within the tentacle base which is contiguous with the pedalium, but it is not clear if these represent an early developmental stage of the larger euryteles that are highly concentrated in the tentacle tips [2]. Studies comparing expression of “venom implicated genes” across medusa body parts can help identify additional putative site(s) of venom production and regions of nematogenesis in cubozoans.

Unique among cnidarians, only cubozoan medusae possess image-forming eyes implicated in visual-guided behavior [8]. Two complex eyes, complete with lens and retina, are located on special sensory structures called rhopalia on each of the four sides of the medusa bell. Each rhopalium also possesses a statocyst (balance organ), and two pairs of ocelli (light receptors) [8, 22] that lack a lens, like other simple animal eyes (having a single pigment cell and at least two photoreceptors [23–25]). Molecular components of the opsin-mediated phototransduction pathway have been identified in the rhopalium of the cubozoans *Tripedalia cystophora* and *C. brevipedalia* (as *Carybdea rastonii*) [26, 27], as well as in non-rhopalium medusa tissue, and planulae, which have simple eye spots [24, 27, 28]. Cubozoan planulae eye spots (ocelli) studied in *T. cystophora* are single cell structures containing a cup of pigment and photosensory microvilli, serving as rhabdomeric photoreceptors [24, 25]. Opsins have also been documented in other cnidarians without lens eyes [29–35] suggesting a role in light perception independent of image formation. Studies comparing expression of “vision implicated genes” across medusa body parts with and without eyes, and planula larvae with simple eyes, can help identify molecular components of the opsin-mediated phototransduction pathway in the rhopalium and aide in discovery of putative areas of extraocular photoreception in cubozoans.

Although most cnidarians reproduce sexually by simple broadcast spawning of their gametes (sperm and/or eggs), many cubozoan species engage in complex sexual behaviors including synchronous spawning aggregations, mating and internal fertilization [1–3]. In species with internal fertilization, such as *Alatina alata* [2] and *Copula sivickisi* [1], sperm are taken up by the female (as a spermatophore in the latter species), and following fertilization blastulae or planulae are released into the water [7, 36]. Histological studies have detected a gametogenic differentiation gradient within the gonads of two cubozoan taxa (*Copula sivickisi* and *Carybdea xaymacana*) [1, 22, 37], but it is unknown how widespread this process is in cubozoans. Equally elusive is the location of fertilization in cubozoans, although it has been hypothesized to occur in the gastrovascular cavity adjacent to the ovaries in a few species [1, 2]. Comparing expression patterns of “sex implicated genes” in different body parts can help determine whether a gametogenic differentiation gradient is present in additional cubozoan species, and might also aide in pinpointing more precisely the site of fertilization.

The goals of this study were to identify candidate genes in box jellyfish that may be involved in nematogenesis and venom production, vision and extraocular sensory perception, and sexual reproduction, which for brevity we refer to as “venom”, “vision” and “sex” implicated genes. We focused on the species *Alatina alata,* which provides a number of advantages for molecular investigation of these traits. The distribution of nematocysts has been well-documented in this species [2, 38, 39], and its sting is potent, causing serious human envenomation; like other cubozoans it has both simple and compound eyes on the medusa rhopalia as well as eye spots in planulae (ciliated swimming larvae); and mature medusae of this species form monthly nearshore spawning aggregations at predictable times (8–10 days after the full moon) in Indo-Pacific and Atlantic localities [2, 40, 41]. *A. alata* medusae have also been documented (as *Carybdea alata*) in the open ocean at great depths [2, 42]. The monthly predictability of mature medusae in nearshore waters [2, 43–45] and the ability to obtain planulae in vitro make *A. alata* a particularly favorable candidate for a cubozoan model.

RNA-Seq transcriptomics provides a reasonably unbiased method of profiling putative candidate genes in different body parts and life stages. This approach has been used successfully in other cnidarians to identify putative genes involved in different stages of a scyphozoan life cycle [46] and in different polyp types in a colony hydrozoan [47]; to identify candidate venom genes in anthozoans [48] and in the tentacles of venomous scyphozoans and cubozoans [5, 19]; and for reconstructing evolutionary relationships within Cnidaria [49]. In the absence of a reference genome for *A. alata*, we generated a *de novo* transcriptome assembly pooled from RNA-Seq data from specific body parts (gastric cirri, tentacle—including the base of the adjoining pedalium, rhopalium, and ovaries) of a female medusa undergoing internal fertilization during a spermcast mating event. We compared these transcripts to known eukaryote gene and protein databases, and identified genes implicated in venom, vision and sex based on homology and tissue-specific gene expression profiles. We also investigated the expression of these candidate genes in planulae. Presented here is the first functionally annotated transcriptome of *A. alata*, which serves as a valuable resource for understanding the molecular underpinnings of cubozoan biological processes and their mediation of complex behaviors.

## Results

### Sample collection

**Fig. 1:**
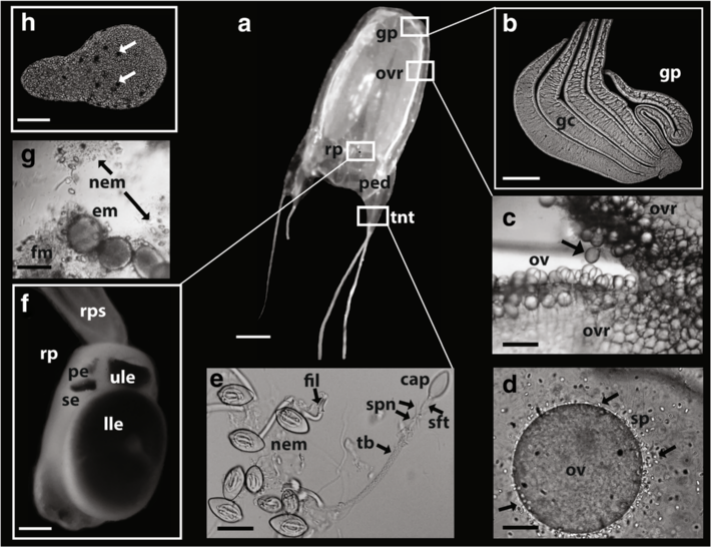
**a**-**h** Morphology of *A. alata* mature female medusa similar to that collected and subsampled for *de novo* transcriptome assembly in this study. White boxes correspond to the location of medusa body parts sub-sampled. **a** Mature *A. alata* medusa (live). **b** A portion of a gastric phacella removed from a live medusa, with five individual gastric cirri. **c** Ovulation documented within the female gastrovascular cavity; arrow indicates imminent release of teardrop shaped ovum. **d** Interaction between recently ovulated egg and spermatozoa (arrows) in the fluid examined from the gastrovascular cavity (representing putative fertilization). **e** Intact nematocysts (euryteles with associated filaments) on the left, and a discharged eurytele on the right, isolated from the tentacle. **f** Rhopalium connected to the rhopaliar stalk showing upper and lower lens eyes, and lateral pit and slit eyes (one of each pair visible). **g** Bundles of blastulae released by females entangled in fibrous material and intact eurytele nematocysts. **h** Swimming planula (within 20 h of blastula release from the female); arrows indicating planulae eyes spots. Abbreviations: cap = capsule of nematocyst; em = embryos; fil = filaments; fm = fibrous material, gc = gastric cirri; gp = gastric phacella (comprised of numerous gastric cirri); nem = nematocyst (birhopaloids)s; ov = ovum (ova); ovr = ovaries; pe = pit eye; ped = pedalium; rp = rhopalium; rps = rhopaliar stalk; se = slit eye; sp = sperm; sft = shaft of nematocyst tubule; spn = spines of nematocyst shaft; tb = tubule of discharged nematocyst; tnt = tentacle. Scale bars: a = ~15 mm, b = ~0.5 mm, c = ~250 μm, d = ~30 μm, e = ~20 μm, f = ~0.2 mm, g = ~100 μm, h = ~30 μm

*Alatina alata* material was collected during a spermcasting aggregation in Bonaire, The Netherlands (April 23-25, 2014, 22:00-01:00) according to the methods in [2]. A single ovulating female medusa (Fig. 1a) was dissected to provide four tissue samples, namely: i. gastric cirri (Fig. 1b), ii. ovaries (Fig. 1c) (within gastrovascular cavity filled with sperm (Fig. 1d)), iii. tentacle (containing nematocysts Fig. 1e) and adjoining pedalium base, which we refer to collectively as the “tentacle sample” below (Fig. 1a), and iv. rhopalium (including rhopaliar stalk) (Fig. 1f). A fifth sample consisted of planulae (Fig. 1h) that developed from blastulae (Fig. 1g) released by females in the lab. Transcripts putatively involved in venom were characterized through their apparent up-regulation in the tentacle and gastric cirri samples; transcripts putatively involved in vision were targeted through analysis of the rhopalium sample; and transcripts putatively involved in sex and embryogenesis were investigated through analysis of the ovaries and planulae samples. We sought to identify the possible onset of expression of candidate genes in the larval planula stage. Given the microscopic size (~150 μm) of the planulae, multiple individuals were pooled to obtain sufficient tissue for RNA isolation and sequencing. Detailed protocol is provided in Methods.

### RNA-Seq and bioinformatics

#### *De novo* transcriptome assembly

**Fig. 2:**
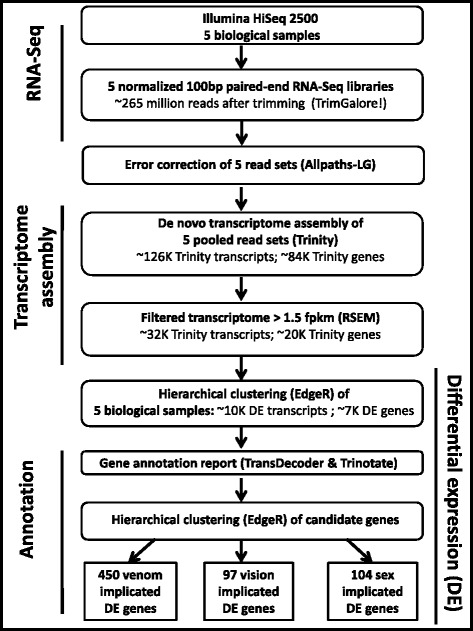
Flowchart of methodology used in transcriptome assembly, gene annotation and differential expression of the five *A. alata* samples analyzed in this study. Additional details provided in Results and Methods. Figure modelled after [47]

RNA-Seq was performed on five different tissues using the Illumina HiSeq2500 Sequencing System (see Methods). Using Trinity software [50, 51] the ~264 million trimmed raw paired-end sequence reads were assembled *de novo* into a pooled transcriptome yielding ~126 KTrinity transcripts corresponding to ~84 K Trinity genes with an N50 of 1994 (Table 1). Throughout this paper, we use the term “*A. alata* gene” to refer to each transcriptome component represented by a unique Trinity gene id, and the term “*A. alata* transcript” to refer to additional transcriptome components that Trinity assigned as multiple putative “isoforms” of a single unique gene id [50, 51]. Transcriptome completeness was assessed using the subset of 248 widely conserved eukaryote core genes (with low frequency of gene family expansion) using the program CEGMA (Core Eukaryotic Genes Mapping Approach) [52, 53]. We retrieved 242 complete CEGs (98 %) and an additional three partial CEGs (1 %), resulting in 99 % CEG representation. We sought to identify highly expressed transcripts which, by definition, are represented by more reads (than lowly expressed transcripts), and thus have a better chance of being contiguously assembled. Therefore we generated a filtered transcriptome comprising a subset of transcripts expressed above a minimum threshold of 1.5 fragments per kilobase per million fragments mapped (fpkm) [46], based on read quantification and alignment accuracy using RSEM [54]. This filtering step is consistent with our aim of identifying highly expressed transcripts for potential candidate genes across different samples types. The filtered transcriptome, hereafter referred to simply as the *A. alata* transcriptome, yielded ~32 K transcripts corresponding to ~20 K genes; N50 = 2545 (Table 1). The percentage of sequences above 1000 bp doubled and the percentage of short genes (200–500 bp) was reduced by half (Additional file 1). Figure 2 illustrates the workflow used in this study (modelled after [47]).

**Table 1:** *A. alata* pooled transcriptome assembly statistics

	Whole transcriptome	Filtered transcriptome (fpkm = 1.5)
No. of transcripts	126,484	31,776
No. of genes	84,124	20,173
Total assembled bases (bp)	125,647,941	48,556,932
Avg (mean) transcript length (bp)	993	1528
Median transcript length (bp)	456	989
Max transcript length (bp)	9993	9993
N50	1994	2545
GC content (%)	39	40
Percent proper pairs	77	78
Samtools percent mapped and paired	75	77

*De novo assembly* began with 264,505,922 trimmed raw reads generated from medusa gastric cirri, ovaries, tentacle (with pedalium base), rhopalium, and planulae samples

#### Functional annotation

Our first objective was to annotate the *A. alata* transcriptome. The longest open reading frames (ORFs) were predicted for transcripts using TransDecoder [50] and subsequently annotated with Trinotate [51], which compiles results of homology searches of reliable databases (i.e., Uniprot; NCBI; eggNOG/GO; HMMER/PFAM, SingalP) to capture Basic Local Alignment Search Tool (BLAST) protein and gene homologies. The resulting Trinotate report for the ~32 K *A. alata* transcripts contained 12,317 BLASTP top hits from TrEMBL and 10,627 BLASTP top hits from SwissProt, from which 656 candidate genes were examined in this study for their putative roles in venom, vision and sex (see Candidate gene profiling below). In total 96 of the top 100 most abundant genes in the transcriptome (based on normalized counts) were assigned at least one Trinotate annotation category: 85 % of those had BLAST top hits; and 63 % corresponded to candidate genes we explored as implicated in either venom, vision or sex in this study (Additional file 2). The Trinotate report listed 14,551 transcripts corresponding to peptides based on TransDecoder predicted ORFs; 2098 transcripts with transmembrane protein domains (TMHMM database); 1610 transcripts containing the classical secretory signal peptide (SignalP database); and 5252 TrEMBL BLASTP top hits corresponded to cnidarian proteins (Additional file 3).

#### Gene expression patterns and profiles

We then sought to detect gene expression patterns across the five samples (gastric cirri, ovaries, tentacle with pedalium base, rhopalium, and planulae) with the aim of providing a descriptive analysis of the top expressed gene clusters by sample. In order to estimate transcript abundance we aligned each set of reads back to the *A. alata* transcriptome and generated an RNA-Seq fragment counts matrix for each sample using RSEM [54]. We subsequently identified differentially expressed genes (see Methods) which we clustered according to their expression profiles using hierarchical clustering analyses within the framework of the EdgeR Bioconductor software package [55], a preferred methodology for studies lacking biological replicates [46, 56]. Of the ~32 K Trinity transcripts (~21 K Trinity genes) identified in the *A. alata* transcriptome ~10 K transcripts (6676 genes) were found to be differentially expressed, within a broad range, across the five samples (Additional file 4). EdgeR takes the normalized gene counts for all samples (generated from the initial RSEM counts matrix), and then clusters genes with similar mean expression rates across samples [56]. Gene clusters are visualized in the form of a heatmap, permitting pinpointing of genes abundant in certain samples that might be of interest as candidate genes. The results of hierarchical clustering were consistent with our initial RSEM evaluation of abundant genes by sample.

**Fig. 3:**
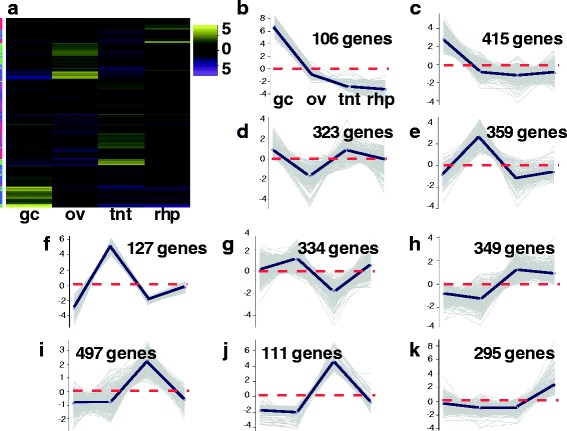
**a**-**k** Heatmap of *A. alata* medusa samples. Hierarchical clustering (EdgeR) and corresponding ten subcluster profiles for the 2916 genes differentially expressed across *A. alata* medusa samples (gastric cirri, ovaries, tentacle (with pedalium base), rhopalium) (of the identified ~20 K Trinity genes. Intensity of color indicates expression levels for each of the ten hierarchical clusters (vertical access). Bright yellow patches correspond to the highest peaks for each k-mean subcluster profile. K-mean profiles (**b**-**k**) match the order of column names in **a**, representing the mean expression of gene clusters highly abundant in each sample (centroid demarcated by the solid line; zero indicated by the horizontal dashed red line). Three bright yellow transcript clusters in the gastric cirri column correspond to each of the peaks seen in plots **b**, **c** and **d** in the ovaries column correspond to plots **e**, **f**, **g** and in the tentacle column correspond to **h**, **i**, **j**, while the two bright yellow clusters in the rhopalium column correspond to peaks in plots **k**, and to the less prominent peaks in plots **g** and **h**. The vertical colored bar on the left of the heatmap (**a**) indicates distinct patterns corresponding to the ten subcluster profiles (sc = subcluster number), for which the number of genes each comprises is indicated. Abbreviations: gc = gastric cirri, ov = ovaries, tnt = tentacle (and pedalium base), rhp = rhopalium, and pln = planulae. (Original matrix in Additional file 5)

In an attempt to identify genes that were specifically abundant in each medusa sample, we conducted subsequent hierarchical clustering with the planulae sample excluded. EdgeR analyses of genes from just the four medusa samples (gastric cirri, ovaries, tentacle (with pedalium base) and rhopalium) revealed 2916 differentially expressed genes. To identify patterns of highly expressed gene clusters by medusa sample, we generated a heatmap of the subset of 2916 genes and further partitioned the subset into 10 gene subcluster profiles based on mean expression patterns across samples (Fig. 3a-k; Additional file 5). Furthermore, redoing the hierarchical clustering and subcluster profiling analyses for all five samples using the three subsets of candidate genes (putative venom, vision and sex genes) allowed us to hone in on gene clusters that were relevant to transcriptome functional annotation and profiling of different *A. alata* samples.

#### Tissue-specific “core genes”

**Fig. 4:**
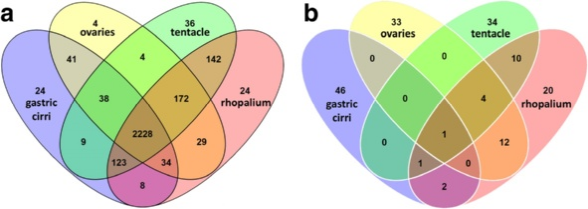
**a**, **b** Venn diagrams showing overlap of genes differentially expressed exclusively in *A. alata* medusa samples (gastric cirri, ovaries, tentacle, rhopalium). **a** Shows that of the 2916 total genes differentially expressed across the four samples 2228 (76 %) are expressed in all samples, 24 are unique to gastric cirri, 4 are unique to ovaries, 38 are unique to the tentacle, and 24 are unique to the rhopalium. **b** Shows that of the top 50 most highly differentially expressed genes by sample type, a single gene is expressed in all four samples. The subset of genes unique to the top 50 most abundant genes by sample, called the “core genes” herein, comprises 46 genes in the gastric cirri, 33 in the ovaries, 34 in the tentacle (and pedalium base), and 20 in the rhopalium. Core gene annotations provided in Additional file 6 and summarized in histograms Fig. 6a-d

**Fig. 5:**
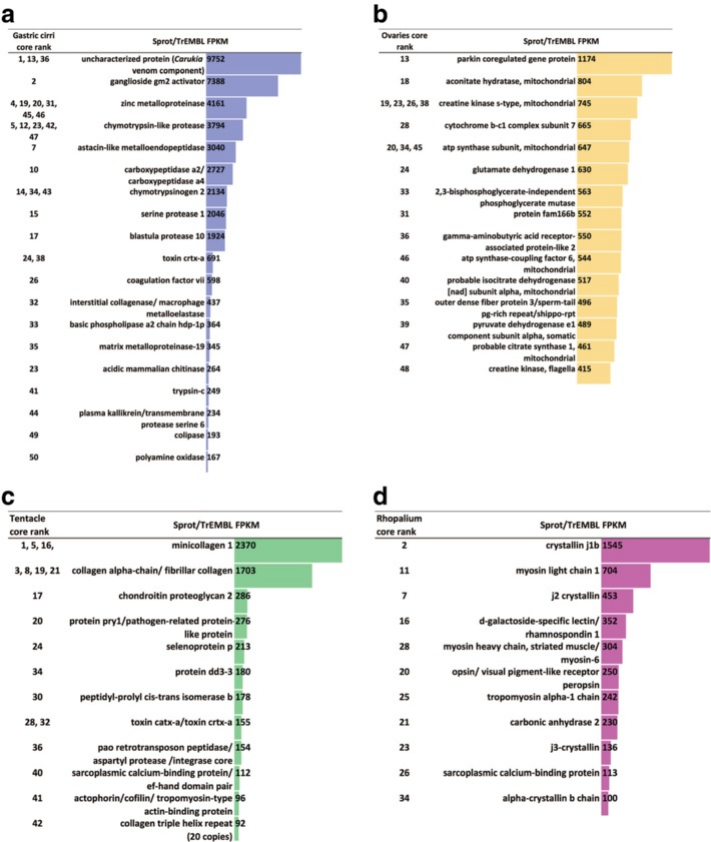
**a**-**d** Abundances of annotated “core genes” in the *A. alata* transcriptome according to medusa sample. Column headings correspond to rank(s) among the top 50 of each core gene (or gene family) by sample according to the Venn diagram in Fig. 4b, protein annotation from UniProtKB SwissProt (Sprot) and TrEMBL (separated by a back slash) and fpkm values in **a** gastric cirri, **b** ovaries, **c** tentacle (and pedalium base), **d** rhopalium. Genes with putative functions in sperm motility are indicated with asterisks (*) in **b**. Genes lacking Trinotate annotations are not included. Detailed statistics (fpkm, counts, DE values for top 50 ranked genes by sample with annotations) provided in Additional file 6)

Our next objective was to identify specific genes that are highly expressed in particular body parts (abundant with respect to other samples), and subsequently identify functional categories informed by the Trinotate report. We constructed a Venn diagram of the 2916 differentially expressed genes from the four medusae samples (Fig. 4a), which revealed that 76 % (2228 genes) were expressed to some degree in all medusa samples (Additional file 1). A subset corresponding to the top 50 most highly expressed genes per sample was plotted in a second Venn diagram (Fig. 4b), and genes unique to each sample’s top 50 were respectively designated as that sample’s “core genes” (Additional file 6). Only one gene (lacking a Trinotate annotation) among each sample’s top 50 most highly expressed genes overlapped in all four samples. An assessment of the core genes revealed that only about 40 % had Trinotate-generated annotations (Fig. 5a-d). Gastric cirri core genes (n = 46) corresponded mostly to putative proteins (26 annotations) implicated in venom and digestion, including metalloproteinases (Fig. 5a) [5, 57, 58]; ovaries core genes (n = 33) corresponded to putative proteins (22 annotations) involved in gametogenesis, including Vitellogenins (Fig. 5b), which, although well studied in bilaterians [59], are newly reported in cubozoans herein; tentacle core genes (n = 34) corresponded to many putative proteins (19 annotations) associated with nematocyst development, including minicollagens (Fig. 5c) [60–62]; and rhopalium core genes (n = 20) corresponded to several putative proteins (12 annotations) identified in vision and the phototransduction pathway, including J-crystallins (Fig. 5d) [28, 33, 63].

#### Candidate gene profiling

**Fig. 6:**
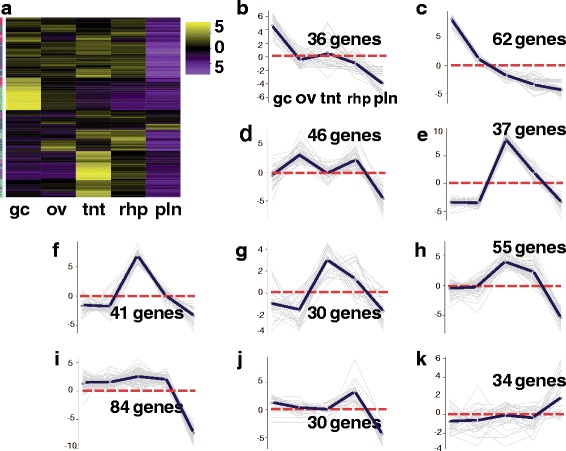
**a**-**k** Venom Heatmap for *A. alata*. Hierarchical clustering (EdgeR) and corresponding ten subcluster profiles for the 455 genes implicated in venom differentially expressed across *A. alata* medusa (gastric cirri, ovaries, tentacle (with pedalium base), rhopalium) and planulae samples. Intensity of color indicates expression levels for each of the ten hierarchical clusters (vertical access). Bright yellow patches correspond to the highest peaks for each k-mean subcluster profile. K-mean profiles (**b**-**k**) match the order of column names in **a**, representing the mean expression of gene clusters highly abundant in each sample (centroid demarcated by the solid line; zero indicated by the horizontal dashed red line). Two bright yellow transcript clusters in the gastric cirri column correspond to peaks in plots **b** and **c**; one cluster in the ovaries column corresponds to plot **d**; four clusters in the tentacle column correspond to plots **e**, **f**, **g** and **h**; one cluster in the rhopalium corresponds to plot **j** and to the less prominent peak seen in plot **i**; one cluster in the planulae column corresponds to plot **k**. The vertical colored bar on the left of the heatmap (**a**) indicates distinct patterns corresponding to the ten subcluster profiles (sc = subcluster number), for which the number of genes each comprises is indicated. Abbreviations: gc = gastric cirri, ov = ovaries, tnt = tentacle (and pedalium base), rhp = rhopalium, and pln = planulae. Gene annotations by subcluster provided in Additional file 7

**Fig. 7:**
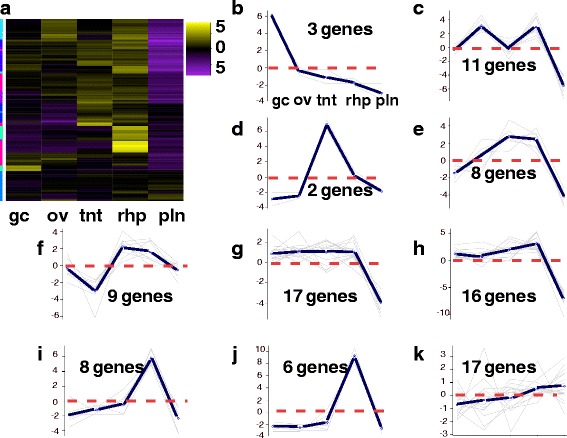
**a**-**k** Vision Heatmap for *A. alata*. Hierarchical clustering (EdgeR) and corresponding ten subcluster profiles for the 97 genes implicated in vision and the phototransduction pathway differentially expressed across *A. alata* medusa (gastric cirri, ovaries, tentacle (with pedalium base), rhopalium) and planulae samples. Intensity of color indicates expression levels for each of the ten hierarchical clusters (vertical access). Bright yellow patches correspond to the highest peaks for each k-mean subcluster profile. K-mean profiles (**b**-**k**) match the order of column names in **a**, representing the mean expression of gene clusters highly abundant in each sample (centroid demarcated by the solid line; zero indicated by the horizontal dashed red line). One bright yellow transcript clusters in the gastric cirri column correspond to a peak in plot **b**; one cluster in the ovaries column corresponds to plot **c**; three clusters in the tentacle column correspond to plots **d**, **e** and **f**; three bright yellow clusters in the rhopalium column correspond to peaks in plots **h**, **i** and **j**, and two less intense clusters correspond to peaks in plots **g** and **k**, and a slightly intense yellow gene cluster in the planulae column corresponds to the peak in plot **k**. The vertical colored bar on the left of the heatmap (**a**) indicates distinct patterns corresponding to the ten subcluster profiles (sc = subcluster number), for which the number of genes each comprises is indicated. Abbreviations: gc = gastric cirri, ov = ovaries, tnt = tentacle (and pedalium base), rhp = rhopalium, and pln = planulae. Gene annotations by subcluster provided in Additional file 8

**Fig. 8:**
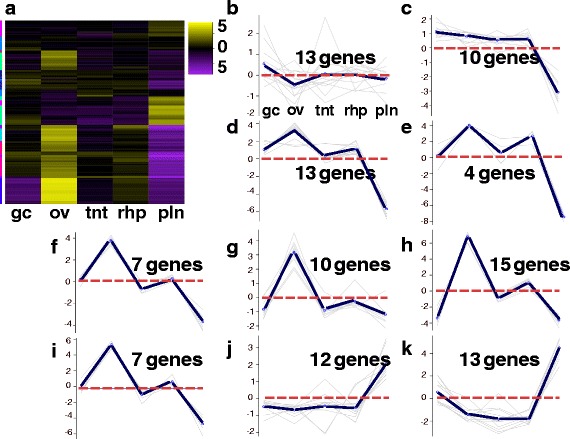
**a**-**k** Sex Heatmap for *A. alata*. Hierarchical clustering (EdgeR) and corresponding ten subcluster profiles for the 104 genes implicated in sex and early development differentially expressed across *A. alata* medusa (gastric cirri, ovaries, tentacle (and pedalium base), rhopalium) and planulae samples. Intensity of color indicates expression levels for each of the ten hierarchical clusters (vertical access). Bright yellow patches correspond to the highest peaks for each k-mean subcluster profile. K-mean profiles (**b**-**k**) match the order of column names in **a**, representing the mean expression of gene clusters highly abundant in each sample (centroid demarcated by the solid line; zero indicated by the horizontal dashed red line). Two yellow gene clusters in the gastric cirri column correspond to peaks in plot **b** and **c**; six bright yellow clusters in the ovaries column corresponds to profiles **d**-**i**; no major abundant gene clusters were detected in the tentacle column; four less intense clusters in the rhopalium column correspond to peaks in plots **b**, **d**, **e**, and **h**; two bright clusters in the planulae column correspond to peaks in the subcluster profiles **j** and **k**. The vertical colored bar on the left of the heatmap (**a**) indicates distinct patterns corresponding to the ten subcluster profiles (sc = subcluster number), for which the number of genes each comprises is indicated. Abbreviations: gc = gastric cirri, ov = ovaries, tnt = tentacle (and pedalium base), rhp = rhopalium, and pln = planulae. Gene annotations by subcluster provided in Additional file 9

Using the tissue-specific core gene annotations (see above) as a starting point for identifying candidate genes in *A. alata* medusa body parts, we compiled three candidate-gene lists comprising 148, 109 and 39 terms (related to genes and/or gene functions) broadly associated with “venom”, “vision”, and “sex”, informed primarily by relevant genes and proteins documented in the scientific literature (Additional files 7, 8 and 9: provides lists of terms, references and differential expression matrices for all putative candidate genes). Subsequently, we queried the *A. alata* Trinotate report separately using each of the lists to identify matching terms among BLASTX, BLASTP and PFAM (protein family) top hits from the Trinotate transcriptome annotation report. This generated three additional targeted Trinotate annotation reports consisting of gene subsets we categorized as “venom implicated genes” (Additional file 7), “vision implicated genes” (Additional file 8), and “sex implicated genes” (Additional file 9). Subsequently, EdgeR hierarchical clustering (see above) was conducted separately on each subset of corresponding candidate genes, generating three new heat maps (Figs. 6, 7 and 8); profiling the respective patterns of expression of each set of candidate genes across all five samples. In all cases, gene cluster patterns were divided into 10 subclusters based on mean expression patterns for genes with potential association with venom (*n* = 450) (Fig. 6b-k); vision (*n* = 97) (Fig. 7b-k); and sex (*n* = 104) (Fig. 8b-k). By comparing planulae samples with samples from body parts of the mature female medusa, an additional aim of this study was to detect the potential onset of expression of these possible candidate genes within developing planulae. Differential expression matrices for all candidate genes and their respective annotations are provided in Additional files 7, 8 and 9. We did not detect expression of most candidate genes in the planulae sample. Instead, we found that transcripts most highly expressed in the planulae comprised mainly: histones (core and early embryonic), *nanos* transcription factor, and genes implicated in neurogenesis, mitosis, microtubule, and protein processing (Additional file 10).

##### Putative venom implicated genes

Here we highlight our findings of the 450 transcripts we broadly refer to as “venom implicated genes” based on preliminary candidate gene profiling (above). This effort focused specifically on identifying genes highly expressed in the tentacle (used in prey capture) and gastric cirri (used in digestion). By comparing a body part with tissue abundant in penetrant nematocysts (tentacle and adjoining pedalium base) (Fig. 1e) with one lacking nematocysts (gastric cirri Fig. 1b) our aim was to identify putative site(s) of venom production and nematogenesis in *A. alata*. Although nematocysts are typically abundant in the gastric cirri of many box jellyfish species [15], only a single individual nematocyst has been documented in the gastric cirri of mature *A. alata* medusae [2] despite examination of hundreds of mature specimens in several independent studies [2, 15, 38, 40]. Conversely, nematocysts (Fig. 1e) are primarily concentrated in the tentacle (which is contiguous with the pedalium) of *A. alata* [2], as is the case with all cubozoans [15]. Hierarchical gene cluster profiling (Fig. 6 a-k) revealed that many of the putative venom implicated genes were fittingly highly expressed in the tentacle (Figs. 5c and 6e-i), but were surprisingly also highly abundant in the gastric cirri (Figs. 5a and 6b,c; Additional file 7).

#### CaTX/CrTX toxin family genes

**Fig. 9:**
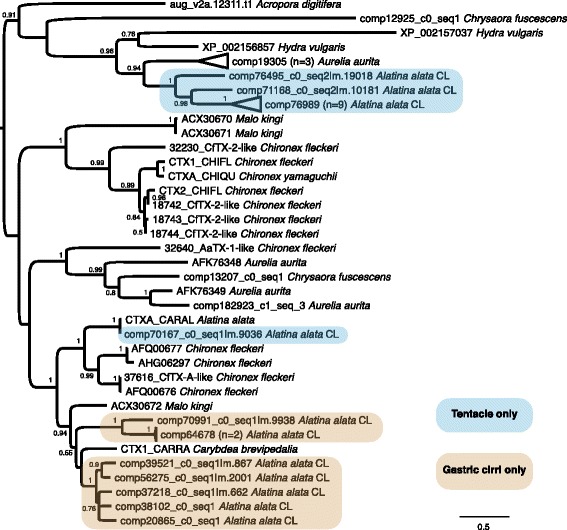
Cnidarian CaTX/CrTX toxin family gene tree. ML topology of all known homologs of the CaTX/CrTX toxin family in cnidarian taxa from NCBI Genbank and transcriptome components of *A. alata* in this study. Assumes the WAG + G + F model of amino acid evolution, as specified as most appropriate by ProtTest v. 3.2. Shimodaira-Hasegawa-like branch support indices are shown at each node. Tissue-specific expression patterns correspond to transcripts primarily enriched in the tentacle (in blue) and gastric cirri (in beige). In *A. alata*, the expression of genes annotated as the CaTX-A homolog is specific to the tentacle (and pedalium base) sample (comp70167, comp71168), while genes annotated as the CrTX-A homolog are specific to gastric cirri and a single tentacle gene (comp76495). Additionally, the expression of a single gene (comp76989) annotated as both CrTX and CaTX is tentacle specific

We identified eleven different homologs of the CaTX/CrTX toxin family (also annotated individually as CrTX-A or CaTX-A). These were either abundant almost exclusively in the tentacle (*n* = 4) (Figs. 5c and 6e, f) or in the gastric cirri (*n* = 7) (Figs. 5a and 6b, c). This gene family consists of pore-forming toxins that cause pain, inflammation and necrosis during human envenomation [64] and prior to this study has exclusively been associated with venom from nematocysts [18, 19, 65]. The taxonomically restricted CaTX/CrTX toxin family [66, 67], previously called the “box jellyfish toxin family” [64, 66, 67], was thought to be restricted to medusozoans [5], but recently a homolog was also identified in the anthozoan coral *Acropora* [68]. Gene tree reconstruction (Fig. 9) of the eleven CaTX/CrTX gene homologs abundant in either the gastric cirri or the tentacle confirmed homology of the *A. alata* transcripts with CaTX/CrTX toxin family genes in other cnidarians [5, 19, 68]. The analysis recovered four well-supported groups of *A. alata* CaTX/CrTX genes, each exclusively containing transcripts with tissue-specific expression patterns, either in tentacle or gastric cirri (Fig. 9). One group includes three *A. alata* homologs (annotated as CaTX/CrTX, CaTX or CrTX) specific to the tentacle that group with several non-cubozoan medusozoans including the coral *Acropora.* Three additional groups are all within a well-supported cluster of CaTX/CrTX genes identified from cubozoan taxa. One of these nested groups includes the homolog (CaTX-A) reported more than a decade ago [65] in *A. alata* (as *Carybdea alata*). Homologs of this gene are sister to a sub-group comprised of *Chironex fleckeri* homologs, which have been identified exclusively from tentacle tissue. The two additional nested groups (Fig. 9) include the homolog (CrTX-A) reported more than a decade ago in *Carybdea brevipedalia* (reported as *C. rastonii*), consisting of transcripts only identified in our gastric cirri sample, in a well-supported cluster of homologs derived from *Carybdea brevipedalia* and *Malo kingi*. Ours is the first report of expression of the CaTX/CrTX toxin family in a medusozoan body part that lacks nematocysts.

#### Venom components

We report that an abundance of “cysteine-rich secretory protein family” (CRISPs) transcripts occurred almost exclusively in either the gastric cirri or the tentacle. Some examples include: “serine protease coagulation factor vii”, “chymotrypsin-like elastase family” homologs, and “serine protease inhibitor” (Figs. 5a and 6c). Likewise, multiple homologs of the “zinc metalloproteinase/astacin (peptidase family m12a)” (Figs. 5a, c and 6b, c, f, i, k) were primiarily abundant in the gastric cirri, but with high expression in the tentacle as well. Zinc metalloproteinases are peptidases with known roles in venom maturation in spiders and snakes, and were recently identified as tentacle venom components of some jellyfish taxa [5, 18, 58]. Conversely, homologs of well-known bilaterian venom proteins (e.g., pit viper (*Croatulus*)/zinc metalloproteinase nas-4/venom factor (Fig. 6i); scorpion (*Lychas*), venom protein 302 (Fig. 6h); the “venom prothrombin activator pseutarin-c non-catalytic subunit” from the eastern brown snake (*Pseudonaja textilis*) (Fig. 6i); and “alpha-2-macroglobulin family N-terminal region” (Fig. 6i) were most abundant in the gastric cirri and tentacle in this study, but were also expressed in the ovaries and rhopalium samples.

#### Nematocyst structural genes

**Fig. 10:**
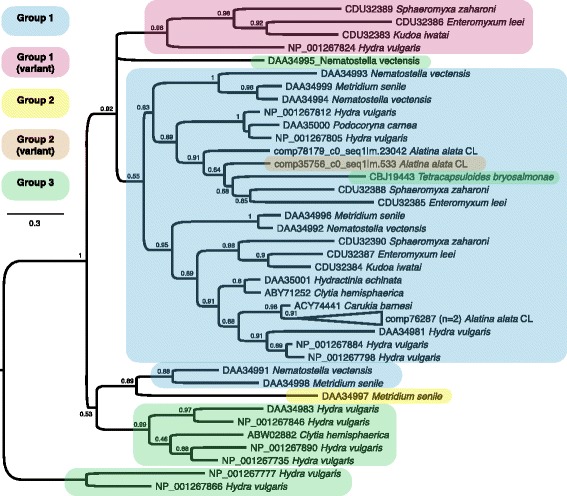
Cnidarian minicollagen gene tree. ML topology of minicollagen gene family in cnidarian taxa from NCBI Genbank and transcriptome components of *A. alata* in this study. Assumes the BLOSS62I-G-F model of amino acid evolution, as specified as most appropriate by ProtTest v. 3.2. Shimodaira-Hasegawa-like branch support indices are shown at each node. All *A. alata* minicollagen types were more closely related to non-cubozoan homologs. Following Shpirer et al. 2014, Group 1 minicollagens possess N- and C-terminal cysteine rich domains (CRDs) of a regular form (CXXXCXXXCXXXCXXXCC). We use the label Group 1 (variant) to identify those minicollagens having three or more regular CRDs. Group 2 minicollagens possess one regular and one irregular CRD, with the variant form having the regular CRD at the C-terminus, whereas Group 3 minicollagens possess irregular CRDs at both termini. One (comp76287_c0) of the three recovered minicollagen homologs from *A. alata* was expressed across all five samples, but most abundant in the tentacle (and pedalium base)

Genes encoding putative nematocyst structural proteins were also characterized in this study. This is in line with our aim to characterize molecular components of cubozoan nematocysts and pinpoint putative regions of nematogenesis, given the current view that venom deployment in medusozoans is exclusively controlled by nematocysts. Our findings revealed that three minicollagens, key components in nematocyst capsule development [62], were abundant almost exclusively in the *A. alata* tentacle (with adjoining pedalium base) (Figs. 5c and 6e, h), with slight expression signal in all other medusa samples. All three of the minicollagen genes identified from *A. alata* possess the characteristic collagen-like domain of short repeated tripeptides of the form Gly-X-Y flanked on both sides by proline repeats by N- and C-terminal cysteine rich domains (CRD). The CRDs for two of the three genes are of the regular form (CXXXCXXXCXXXCXXXCC) and thus would be classified as Group 1 minicollagens [69]. The third has a regular CRD at the C-terminus but a variant form at the N-terminus that we refer to as Group 2 variant. Gene tree reconstruction of minicollagen genes for cnidarian taxa (Fig. 10) show that the minicollagens identified for *A. alata* cluster primarily with other Group 1 minicollagens. Of the additional nematogenesis related genes [17, 61, 62, 70–74] expressed in this study, most were almost exclusively abundant in the tentacle (with adjoining pedalium base). They include “nematocyst outer wall antigen” (NOWA) (Fig. 6e), “chondroitin proteoglycan 2” (Fig. 6f), “nematoblast-specific protein nb035-sv2/nb035-sv3/nb012a” (Fig. 6e, f), “nematogalectin-related protein” (Fig. 6e), and “Dickkopf-related protein 3” (Fig. 6e, f, h). Five different “Dickkopf-related protein 3” homologs were abundant in *A. alata* tentacle sample; with only two having notable expression in other body parts (gastric cirri, ovaries or rhopalium).

##### Putative vision implicated genes

Here we highlight our findings of the 97 transcripts we broadly refer to as “vision implicated genes” based on preliminary candidate gene profiling (above). This effort focused specifically on genes expressed in the rhopalium of *A. alata*, which bears a pair of lens eyes with cornea and retina, two pairs of simple ocelli-comprising photoreceptors, and a statocyst (Fig. 1f). By comparing the rhopalium with its visual capabilities and planulae with its known eye-spot photoreceptors, against the medusa samples that lack known photoreceptors (gastric cirri, ovaries and tentacle), our aim was to identify the expression of opsins and other vision implicated genes in the rhopalium of *A. alata*, as well as in putative extraocular photoreceptors in *A. alata*. Hierarchical gene cluster profiling (Fig. 7a–k) revealed that most of the 97 putative vision implicated genes (see “gene-profiling” above) were abundant in the rhopalium (Fig. 5d), but in many cases they were more highly expressed in other samples, in particular in medusa samples (Fig. 7c, e-j; Additional file 8).

#### Opsins

**Fig. 11:**
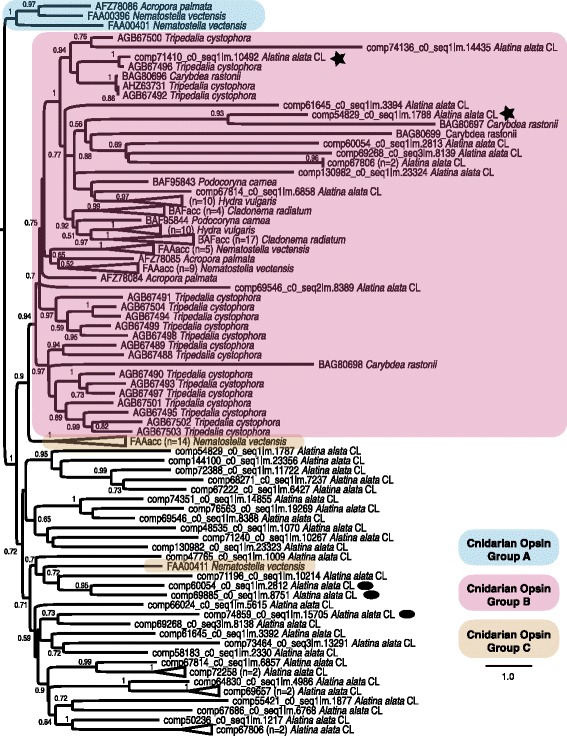
Cnidarian opsin gene tree. ML topology of all known homologs of the opsin gene family in cnidarian taxa from NCBI Genbank and transcriptome components of *A. alata* in this study. Assumes the LG + G model of amino acid evolution, as specified as most appropriate by ProtTest v. 3.2. Shimodaira-Hasegawa-like branch support indices are shown at each node. Blue, pink and brown shading correspond to cnidarian opsin groups A, B and C, respectively, recognized by Feuda et al. (2012). Group A is arbitrarily chosen for rooting the topology; Group B is monophyletic, but Group C cannot be given our derived topology. Stars denote opsin transcripts almost exclusively abundant in the *A. alata* rhopalium sample (comp54829, comp71410) and ovals denote the same in the planulae sample (comp60054, comp69885, comp74859)

In this study the Trinotate report corresponding to the *A. alata* transcriptome contained a total of 41 transcripts with PFAM annotations corresponding to homologs of the “7 transmembrane receptor (rhodopsin family)”. Of the rhodopsin family, opsins are considered universal light sensitive proteins associated with photoreceptor cells of animal retinas. We found eleven opsin homologs to be variably expressed across *A. alata* medusa samples, with only six homologs most abundant in the rhopalium (Figs. 6g, h and 7i, h). A gene tree reconstruction of all rhodopsin family cnidarian genes (Fig. 11), which we rooted on the group that includes all previously known medusozoan opsins, recovered two of the three previously identified cnidarian opsin groups [30]. Group A includes only anthozoan taxa, while Group B includes all opsins previously known from medusozoans, eleven transcripts from *Alatina* and a few from anthozoans. However, cnidarian opsins previously identified as Group C fell into two groups—one of which includes thirty *A. alata* transcripts. This appears to be the first example of medusozoan opsins outside group B cnidarian opsins.

Among the top 10 most abundant genes in the rhopalium of *A. alata* was a homolog of the cubozoan lens-eye opsin for *Carybdea rastonii* (=*C. brevipedalia*) (Figs. 5d and 7i). The *Carybdea* lens eye opsin was also highly expressed in all *A. alata* samples, including planulae which have eye spots (Fig. 1h). Normalized counts revealed three additional opsin genes that were not differentially expressed across all samples, were almost exclusively found in the planulae sample (Fig. 11). Only two *A. alata* putative rhodopsin family homologs were expressed almost exclusively in the rhopalium (Trinotate top BLAST hits: “dopamine receptor 2” and “visual pigment-like receptor peropsin”) (Fig. 11). Among the putative “rhodopsin family” genes expressed in medusa samples, including those annotated in the Trinotate report as non-opsin based photoreceptors, were: “compound eye opsin bcrh1/d(1b) dopamine receptor” (Fig. 7e) and “opsin rh1/mu-type opioid receptor” (Fig. 7h), “blue-sensitive opsin” (Fig. 7k), “visual pigment-like receptor peropsin” (Fig. 7d), “melanopsin-b” (Figs. 7b and 8b; Additional files 8 and 9).

#### Chromophores

We found that several isozymes of *cis*-retinol dehydrogenase, members of the retinoic acid signaling pathway, which convert retinol to retinal (Vitamin A), were expressed in the rhopalium, and in other samples including planulae (Fig. 7c, g, k). In animals, retinal (i.e., 11*-cis-*retinal) is bound to opsin on the photoreceptors of the retina [35], and is thought to be a universal chromophore (light-activated pigment), though various chromophores are used across Metazoa [35]. Carotenoid oxygenase beta, beta-carotene 15,15'-monooxygenase (BCDO1) (Fig. 7g, h) and beta, beta-carotene 9',10'-oxygenase (BCDO2), known to irreversibly cleave carotenoids to produce the essential visual pigments retinal and retinoic acid respectively [75], were expressed in the four *A. alata* medusa samples (Fig. 7e), suggesting that the catalytic components are present to make retinal. We also report the expression of a putative blue-sensitive photoreceptor protein and circadian clock regulator “cryptochrome-1” in the rhopalium, but with high expression in the gastric cirri (Fig. 8b). This suggests the presence of an additional putative chromophore in *A. alata* that functions in extraocular blue-light mediated behaviors (e.g., phototaxis), previously documented in coral and other metazoans [76–79].

#### Crystallins

We found transcripts showing similarity to all three known J-crystallin groups (J1, J2, J3), and all were highly expressed in the rhopalium (Figs. 5d and 7i, j). The J2 crystallin homolog was expressed in all samples including planulae (Fig. 7i); J3 crystallin was almost exclusively expressed in the rhopalium (Fig. 7j); as were all but a single J1 crystallin homolog that was also abundant in the ovaries (Figs. 6d and 7j). Crystallins are water-soluble stable structural proteins that provide transparency and increase the refractive index of eye lenses, though most also have roles unrelated to lens function. Numerous types of crystallins are found across Metazoa, and many are identical (or closely related) to commonly expressed metabolic enzymes or stress proteins [63, 80, 81]. J-crystallins are classified in three evolutionarily independent groups, J1, J2 and J3, and thus far have only been reported in cubozoans [63, 82, 83]. A study on *T. cystophora* showed that the promotors of all three J-crystallin genes can be activated by the paired domain transcription factor PaxB, but the promotor sites are non-homologous among the three J-crystallin types [63]. We aligned all known cubozoan J-crystallins (J1, J2, J3) with the respective *A. alata* homologs identified in this study (Additional file 11). The resulting three alignments illustrate the similarity between *A. alata* transcripts identified in this study and homologs of the three distinct *T. cystophora* J-crystallin types. Additionally, Alpha-crystallin B chain” (vertebrate lens heat-shock proteins) (Fig. 7i) was abundant in the rhopalium, but expressed in all five samples. Conversely, we report transcripts annotated as S-type crystallin (cephalopod lens protein) variably expressed across samples: S-crystallin 2 abundant in the rhopalium and absent in ovaries (Fig. 7f), S-crystallin 3 abundant almost exclusively in the tentacle (Fig. 7d), and S-crystallin 4 most highly expressed in planulae and gastric cirri (Fig. 7k).

#### Homeobox genes and transcription factors

Expression of putative homeobox proteins “Six1b” and “Six4” and the “Six” transcriptional co-activator “eyes absent” (Eya) homolog occurred in all *A. alata* medusa samples, with the highest expression in the gastric cirri. Across Metazoa, the Six-Eya complex functions downstream from certain Pax homeobox genes in a diversity of developmental processes including early eye development [84]. The putative “retinal homeobox proteins rx1b” and “rx3” were expressed in all samples, except for in the ovaries in the case of “rx1b”. Retinal homeobox proteins (“rax” or retina and anterior neural fold homeobox) are essential for early eye-development and in regulation of stem cell proliferation in vertebrates [85], but have not previously been reported in cnidarians.

##### Putative sex and development implicated genes

Here we highlight our findings of the 104 transcripts we broadly refer to as “sex implicated genes” based on preliminary candidate gene profiling (above). This effort focused mainly on genes expressed in the ovaries of *A. alata* during ovulation and internal fertilization. By definition, the ovaries are the site of oogenesis, and are situated within the gastrovascular cavity in *A. alata* [2]. Microscopic examination of the gastrovascular cavity of the female *A. alata* medusa in this study revealed ovulation (Fig. 1c) and internal fertilization (Fig. 1d) occurring within this cavity. By comparing our ovaries sample, which also contained zygotes and embryos, with other body parts predicted to lack reproductive material (gastric cirri, tentacle, rhopalium, and planulae), our aim was to identify genes involved in gametogenesis as well as to determine more precisely the location of internal fertilization within *A. alata*, which we expected would occur adjacent to the ovaries, in the sperm-saturated gastrovascular cavity [2]). Hierarchical gene cluster profiling (Fig. 8a-k) revealed that the highest expression of the 104 putative sex and developmental genes (see “gene-profiling” above) occurred in the ovaries (Fig. 8d-i), but that many were also expressed across all samples (Additional file 9).

#### Oogenesis and embryogenesis

**Fig. 12:**
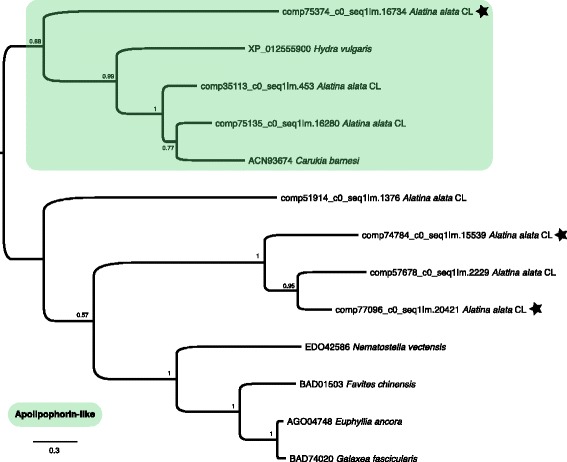
Cnidarian Vitellogenin gene tree. ML topology of all known homologs of the Vitellogenin gene superfamily and apolipophorin-like putative Vitellogenin precursor in cnidarian taxa from NCBI Genbank and transcriptome components of *A. alata* in this study. Assumes the LG + G model of amino acid evolution, as specified as most appropriate by ProtTest v. 3.2. Shimodaira-Hasegawa-like branch support indices are shown at each node. Grey (top) highlights a clade of apolipophorin-like putative Vitellogenin precursor homologs for cubozoan *Carukia barnesi* and *A. alata* from this study and hydrozoan *Hydra vulgaris*, while the non-highlighted clade (bottom) highlights the relationships among known Vitellogenins of cnidarian taxa. Stars designate transcripts most upregulated in the ovaries (comp74784, comp75374 & comp77096); all others are either most highly expressed in the tentacle (comp35113 & 51914) or equally high in the gastric cirri and tentacle samples (comp57678 & comp75135)

We found that homologs for the putative large lipid transfer protein Vitellogenin-2 were most abundant in *A. alata* ovaries (Figs. 6i and 8e), though highly expressed in all medusa samples. Conversely, genes annotated as Vitellogenin-1 or simply Vitellogenin were expressed in all medusa samples, but most abundant in the tentacle (Fig. 6i, g). Likewise, genes annotated as Apolipophorin or Apolipoprotein B-100, the other major animal protein group involved in lipoprotein processing [86], were expressed across all medusa samples but most abundant in the tentacle (Fig. 6g-i). Genes of the Vitellogenin family are responsible for lipid transfer from ovarian follicle cells to oocytes, providing nutrition during embryogenesis in bilaterians and some cnidarians [86, 87], and have a documented role as egg yolk protein precursors in the ovaries of animals, including anthozoans [88]. Gene tree reconstruction of *A. alata* transcripts with known cnidarian Vitellogenin and Apolipophorin-like proteins (Fig. 12) confirmed their homology with other cnidarian large lipid transfer proteins.

We report the abundance of multiple creatine kinase isozymes in the ovaries (Fig. 5b), all of which were expressed to some degree in all samples including planulae (Figs. 6d and 8h). Creatine kinase activity has a documented role in oogenesis and early embryogenesis in mammals [89]. More broadly though, creatine kinase is important in cells with variable rates of energy turnover, such as muscle, neurons, photoreceptors, and primitive spermatozoa [90], which is consistent with its expression in all samples in this study.

#### Sperm motility

Creatine kinase isozymes have also been documented in mediating high energy phosphate transport between sperm mitochondria and sperm flagellar tail [90, 91]. Among the most abundant genes in the ovaries (Fig. 5b) were homologs functioning in sperm tail development and motility: “parkin coregulated gene protein homolog” (Fig. 8i), “outer dense fiber protein 3/sperm-tail pg-rich repeat/shippo-rpt” (Fig. 8h), and multiple putative creatine kinase isoenzymes including “testis isozyme/protoflagellar creatine kinase” (Fig. 8j, h). Likewise multiple putative “serine/threonine-protein kinase” isoenzymes including “testis-specific serine/threonine-protein kinase 1” (Fig. 8g, h) were primarily abundant in the ovaries, but also variably expressed in all five samples. Expression in all samples of these sperm-related genes is consistent with the presence of ubiquitous sperm documented within the female gastrovascular cavity (where the ovaries are located) facilitating internal fertilization in this study (Fig. 1d). Sperm were also abundant in the surrounding seawater, and were undoubtedly adhered to the tentacles and exterior of the medusa bell when all tissue samples were excised from *A. alata*.

#### Sperm capacitation

We report the exclusive upregulation in the ovaries of the putative sperm hyperactivation and acrosomal vesicle reaction promotor protein “cation channel sperm-associated protein” (CatSper2) (Fig. 8g). CatSper genes belong to the family of voltage-gated Ca^2+^ channels that are crucial for sperm fertility in mammals [92]. In particular, capacitation, which typically occurs within the female reproductive tract, involves the destabilization of the acrosomal sperm head membrane allowing greater binding between sperm and oocyte during fertilization due to an increased permeability of Ca^2+^ [92]. Recent studies have showed that sperm of several invertebrate species also undergo capacitation (for a review see [59]). However, until now the possibility of capacitation occurring in sperm of non-bilaterian invertebrates has not been investigated. Unexpectedly, the “CUB and zona pellucida-like domain-containing protein” was most highly abundant in the tentacle and gastric cirri (Fig. 6h, j), despite one of its known roles in ova of attracting sperm to eggs for fertilization in mammals [93]. However, the CUB and zona pellucida-like domain-containing protein is also associated with trypsinogen activation and was previously found in box jellyfish tentacles [5]. Overall, the abundance of transcripts related to sperm dynamics identified in the *A. alata* transcriptome permitted unforeseen profiling of molecular components involved in putative sperm capacitation and fertilization for the first time in a cubozoan.

## Discussion

This study has generated the first annotated transcriptome from multiple tissues of the cubozoan *Alatina alata*, focusing on both the adult (medusa) and larvae (planulae). Our transcriptome significantly adds to the genomic resources available for this emerging cubozoan model. This transcriptome, based primarily on multiple adult body tissues, complements a recently published transcriptome for the same species primarily from early developmental stages [94]. Furthermore, in this study we annotated a large set of genes, allowing for an initial characterization of the molecular complexity of this cubozoan. We also compared transcript abundance across samples to identify genes putatively involved in several key features of cubozoans, namely nematogenesis and venom production, vision and sensory perception, and sexual reproduction. These quantitative data should be considered preliminary, due to lack of replication, but they are suggestive of interesting candidate genes that will be useful for future study. Below we highlight some of the major findings from this initial comparison across samples focusing specifically on genes relevant to i) prey capture and defense, ii) vision and the phototransduction pathway and iii) sexual reproduction and embryogenesis.

### Prey capture and defense

In cubozoans, and more broadly in all cnidarians, prey capture and defense are based on nematocyst (stinging organelles) and associated venom. By comparing a body part abundant in penetrant nematocysts (tentacle and adjoining pedalium base) with one lacking nematocysts (gastric cirri) our aim was to identify putative site(s) of nematocyst development (nematogenesis) and venom production in *A. alata*.

We found that transcripts corresponding to a number of putative nematocyst structural proteins (minicollagens, nematogalectin, NOWA, chondroitin, and Dickkopf homologs) were abundant in the tentacle (and adjoining pedalium base). Although, putative nematogenic transcripts were detected primarily in the tentacle, some were also detected in non-tentacle medusa samples. We expect that this signal stems from the abundant adherent nematocysts covering the medusa bell. Together these findings are consistent with nematogenesis in *A. alata* occurring primarily, but not solely, in the region comprising the tentacle and adjacent pedalium base. Future *in situ* hybridization studies employing genes identified in nematogenesis in this study can help pinpoint more precisely nematogenic regions in *A. alata*.

Venom is a complex cocktail of bioactive compounds (e.g., protein and/or peptides called toxins, salts and neurotransmitters) secreted by one animal that is delivered to another animal by an infliction [57, 95]. Venom disrupts physiological and biochemical molecules of prey and predators, thus facilitating feeding and defense [57]. Nematocysts have long been considered the sole secretory structure for venom deployment in cnidarians [96]. However, we found preliminary evidence for venom production in the gastric cirri, where nematocsyts are lacking in mature *A. alata*. Futhermore, we found that the gastric cirri and tentacle express distinct groups of homologs of a major family of cnidarian venom proteins, the CaTX/CrTX toxin family [66, 67]. This suggests that venom plays an important, and possibly different role in the gastric cirri and tentacle. Venom components likley differ between the nematocyst-bearing tentacle, with a primary role in immobilizing prey and warding off predators, and the gastri cirri, with a primary role in killing and digesting prey [13].

Based on our findings, we hypothesize that *A. alata* has gland cells that secrete toxins associated with the gastric cirri. Evidence was recently presented for toxin-secreting gland cells in the ectoderm of the sea anemone *Nematostella* in regions containing nematocysts as well as areas that may lack nematocysts [97, 98], but our findings represent the first putative case in a cubozoan. Future morphological studies examining the ultrastructure of the stomach and gastric cirri, and venom gene candidate localization studies, will permit testing of the hypothesis of toxin-secreting gland cells associated with the gastric cirri of *A. alata*.

Although differences exist in the exact complement of putative bioactive toxins between the gastric cirri and tentacle sample, the venom cocktail in each body part includes transcripts from similar digestive enzyme families. A recent review of jellyfish toxins lists a number of toxin-like digestive enzymes that are deployed as components of nematocyst venom to disable homeostatic processes in prey or predators [58], as has been noted in animals possessing venom glands [57, 99, 100]. These bioactive proteins function in cytolytic, paralytic and hemolytic roles, thereby facilitating prey digestion [58, 64, 101]. Specifically we note the abundance of several enzyme groups primarily in either the tentacle or gastric cirri in *A. alata* that have been well studied in venomous animals [57, 99, 102, 103], namely astacin-like metalloproteinase and serine proteinase (and inhibitors), and more broadly cysteine-rich secretory proteins (CRISPs). Metalloproteinase and serine proteinase (and inhibitors) are a common component of the venom of animals with venom glands either activating toxins or acting as toxins themselves [18, 99, 100]. In particular, cysteine-rich secretory proteins identified in snake venoms are thought to inhibit smooth muscle contraction in bite victims [104]. Both metalloproteinase and CRISPs have previously been characterized in the tentacles of cubozoan [5, 105] and other cnidarians [19, 20, 61, 68, 106]. The abundance of multiple isozymes of astacin-like metalloproteinase and serine proteinase (and inhibitors) and CRISPs in the gastric cirri and tentacle of *A. alata* suggest a dual role in venom and digestion. Further studies are required to test this hypothesis given the broad involvement of these bioactive proteins in other biological processes [5, 103].

### Vision and the phototransduction pathway

Cubozoans are the earliest diverging animal clade to have image-forming lens eyes, which are part of specialized sensory organs called rhopalia. By comparing a medusa body part bearing conspicuous eyes (the rhopalium) and planulae with eye spots (rhabdomeric photoreceptors) against the medusa samples that lack documented photoreceptors (gastric cirri, ovaries and tentacle), our aim was to profile the molecular components of the opsin-regulated phototransduction pathway and identify additional regions of putative extraocular sensory perception in *A. alata*.

We found that many genes with conserved roles in vision (opsins and crystallins) were abundant in the rhopalium. Although transcripts with putative roles in light-mediated phototransduction pathway were detected primarily in the rhopalium where eyes are present, their expression was broadly detected across the medusa samples, and in some cases in planulae. We expect this signal stems from the presence of additional photoreceptors (yet undescribed) throughout the body of this cubozoan. Together these findings are consistent with a vision-related role for opsins and crystallins in the lens-eye of the rhopalium, as well as a role in putative photoreceptors within non-rhopalium tissues and in planulae eye spots.

The animal phototransduction pathway is mediated by photopigments in photoreceptors consisting of two parts: a membrane protein (apoprotein) “opsin” and a chromophore “retinal” (vitamin A derivative) [107]. Opsins mediate light as phototypical G protein-coupled receptors in both visual and non-visual systems [108]. Currently more than 1000 types of opsin are known across Metazoa, with three subfamilies recognized in bilaterians: rhabdomeric (r-opsins), Go-coupled plus retinochrome retinal G protein-coupled receptor (Go/RGR) and ciliary (c-opsins) [107]. Studies characterizing opsins in cnidarians have raised the possibility that cnidarian opsins form a monophyletic clade referred to as “cnidops” that is sister to the c-opsins [26–28, 109, 110]. Other metazoan-wide analyses of opsins have categorized cnidarian opsins into three groups, A, B and C, in which each of these cnidarians opsin groups has been found, albeit with limited support, to be sister to each of the respective bilaterian opsin groups [30]. One study has revealed support for r-opsins in cnidarians [111], which is consistent with the identification of planulae eye spots as rhabdomeric photoreceptors [24, 25]. Although a consensus is lacking on the relationships between cnidarian opsins and other metazoan opsins, our study identified a number of transcripts with molecular characters corresponding to diverse metazoan opsins (rhodopsin family) in *A. alata*, adding to the known diversity of this gene family within cnidarians.

Our opsin gene tree only included the known cnidarian opsins and thus does not address the question of cnidarian opsin monophyly. However, our analysis recovered homologs within two of the three previously identified cnidarian opsin groups, namely group A and B [30]. Our analysis also recovered many opsin homologs within a large group that also contains cnidarian opsin group C, previously thought to only be present in anthozoans. The presence of this opsin group in both anthozoans and medusozoans suggests that it was present in the cnidarian ancestor.

We found several opsin genes in *A. alata* to be highly expressed in samples other than the rhopalium, and similar results have been reported for opsins in another cubozoan, *T. cystophora* [27, 28]. Based on these findings we hypothesize that cubozoans have opsin-mediated extraocular photoreception activity possibly related to phototaxis, circadian rhythm or light-mediated spawning, such as has been demonstrated in other animals, including anthozoans [29, 35, 76, 112]. These findings are also suggestive of extraocular photosensitivity [25, 108, 110, 113] that has a documented role in rhythmic behaviors and physiological processes in vertebrates and invertebrates, including nematocyst firing in cnidarians [28, 30, 32, 110, 111, 113]. Such suggested extraocular photoreceptor cells may also comprise anatomically dispersed light sensitive neurons, in addition to ciliary or rhabdomeric morphotypes, possibly functioning in dispersed photoreception, also called the “dermal light sense” (for a review see [114]). Future characterization of the absorbance spectra for different opsin types in cubozoans, and visualization of the precise locality of expression using *in situ* hybridization, will help elucidate their potential functions in different medusa body parts and planulae.

In this study the expression of some of the components of the retinal photoisomerization pathway in all samples including planulae suggests that *A. alata* metabolizes the universal chromophore retinal [35, 107]. However, transcripts for a putative blue-sensitive photoreceptor protein and circadian clock regulator cryptochrome homolog suggest an additional putative chromophore in *A. alata* that might function in non-rhopalium related blue-light mediated processes (e.g., phototaxis); such a function has previously been documented in other metazoans [35, 76]. Determining the precise chromophore utilized by *A. alata* must await future functional studies.

Crystallins are multifunctional proteins often related to stress or metabolic enzymes that serve as important lens components controlling optical properties [115]. The dual role crystallins play in eye lens as well as non-eye related tissues is known as “gene sharing” [80]. We found that all three types of J-crystallins previously reported in *T. cystophora* [115] were present in *A. alata* and that these were typically most highly expressed in the rhopalium, with J2 crystallins showing more variable expression across samples.

We also identified transcripts corresponding to the developmental transcription factors Six and eyes absent (Eya), representing the first homologs of these genes identified from cubozoans. Genes in the Six-Eya homolog complex have known functions in eye development, including during embryogenesis and regeneration, in both non-bilaterians and bilaterians [79, 84, 115]. Six-Eya complex genes have been shown to act downstream of Pax genes [84], and PaxB expression has been reported in both adult and larval eyes of *T. cystophora*, where it is inferred to promote J-crystallin expression [28]. We also identified transcripts corresponding to the developmental transcription factors Six and eyes absent (Eya), representing the first homologs of these genes identified from cubozoans. Genes in the Six-Eya homolog complex have known functions in eye development, including during embryogenesis and regeneration, in both non-bilaterians and bilaterians [79, 84, 115]. Six-Eya complex genes have been shown to act downstream of Pax genes [84], and PaxB expression has been reported in both adult and larval eyes of *T. cystophora*, where it is inferred to promote J-crystallin expression [28]. Conversely, in the scyphozoan *Aurelia*, development of simple eyes is mediated by Six-Eya complex genes independent of PaxB expression [116]. Although the Trinotate report for the filtered *A. alata* transcriptome did not contain any transcripts annotated as PaxB, we identified two transcripts (comp95018 and comp20156) annotated as other homeobox genes that appear to be putative PaxB homologs based on sequence identity (tBLASTx) with *Nematostella vectensis* PaxB mRNA. Whether eye development and Six-Eya expression in *A. alata* are dependent or independent of PaxB expression remain open questions. Future studies determining the spatial localization of gene expression during eye development in *A. alata* may be useful for further elucidating the gene regulatory networks functioning in eye development in cubozoan rhopalia and planulae eyes spots.

### Sexual reproduction and embryogenesis

Cubozoan lifecycles alternate between an asexually reproducing sessile polyp stage and a sexually reproducing motile medusa stage. By profiling the transcripts from an adult body part (ovaries) abundant in developing oocytes, our aim was to characterize the molecular components of oogenesis and early embryogenesis in *A. alata*. Additionally, because we found that sperm are internalized and interact with newly ovulated eggs within the gastrovascular cavity of *A. alata* females, our ovaries tissue sample also provided the opportunity to identify genes that might be involved in fertilization.

We identified several apparent homologs of Vitellogenin and Apolipoprotein, which have documented roles in oogenesis and embryogenesis, [86–89] and found these to be most abundant in the ovaries of *A. alata*. Vitellogenin is an animal egg yolk protein that is synthesized in somatic cell lineages and subsequently incorporated into developing oocytes (by receptor mediated endocytosis), eventually serving as a nutrition source during embryogenesis [86–88]. In medusozoans little is known about the characteristics of Vitellogenins as they have only been documented as egg yolk proteins in two coral species [88, 116] and the model sea anemone *Nematostella vectensis* [87]. Vitellogenin proteins are expressed in both ovarian (or putative ovaries in anthozoans, e.g., [87]) and extra-ovarian somatic cells, consistent with their important roles in processing large lipoproteins in a broad range of complex biological processes among metazoans [86], including their distinct role as honey bee venom allergens [117]. Consistent with this, we found that in *A. alata*, apparent homologs of Vitellogenin-2 were expressed most highly in the ovaries, yet they and other Vitellogenins and Apolipoprotein-like homologs were detected in all medusa samples. In this study we also found a number of creatine kinase genes to be most abundant in the ovaries, but many were also detected (though at much lower expression levels) in all of our samples. Creatine kinases play an important role in oogenesis and early embryogenesis in mammals [89], having a broad enzymatic function in yielding ATP by catalyzing the reversible transfer of phosphate from creatine phosphate to ADP in cells with high activity (e.g., photoreceptors, primitive-type spermatozoa) [90, 91].

We did not recover any genes characteristic of meiosis in the ovaries sample of *A. alata,* suggesting that the tissue was composed exclusively of mature ova at the time of sampling. It is also possible that the expression levels of putative meiosis transcripts were too low to be detected by our analyses, given our conservative transcriptome analysis protocol (see Methods). However, few studies exist that characterize the molecular aspects of sexual reproduction in cnidarians [88, 116], limiting the number of potential gametogenic candidate genes targeted in this study. Future transcriptome and proteome profiling studies of the gonads of *A. alata* and other cubozoans during medusa maturation are needed to shed light on the molecular underpinnings of the processes controlling gametogenesis in cubozoans.

In this study we also detected the expression of genes with putative roles in sperm flagella activation, pro-acrosomal vesicles and sperm capacitation, with many of these being most abundant in the ovaries of *A. alata*. These morphological and biochemical changes to the sperm are necessary for the sperm to reach and fertilize an oocyte, and their occurrence has been documented within the female reproductive tract in many animals [92]. Sperm capacitation was previously thought to occur exclusively in mammals, but more recently it has been documented in several invertebrates [59]. Our study is the first to suggest that sperm capacitation might occur within the gastrovascular cavity (putative female reproductive tract) of a cnidarian. We note that although sperm storage structures have been reported in a single family of cubozoans (Tripedaliidae) [1, 118], we do not expect sperm storage to occur in *A. alata*. Morphological observations during the course of this study as well as previous studies in *A. alata* have identified no structure(s) with a putative role in sperm storage in either male or female medusae [2, 38, 119]. Future histological studies of *A. alata* medusae undergoing internal fertilization should elucidate the ultrastructure of the female reproductive tract and provide further insight into fertilization dynamics in this species.

Based on our observations in this and a prior study [2], monthly spermcasting aggregations of *A. alata* medusae consist entirely of males and females with mature gonad morphology. We therefore hypothesize that gonad development occurs offshore in response to environmental and molecular cues related to the lunar cycle that may instigate inshore migrations. During these monthly nearshore aggregations, which span three to four consecutive days, both sexes exhaust their entire gamete reserves in a process known as “controlled gonad rupture” [2, 120]. Male gonads completely disintegrate over the course of several hours, and females simultaneously ingest massive quantities of sperm for internal fertilization. The interaction of sperm and eggs witnessed in the gastrovascular cavity, followed by release of blastulae into the surrounding water by females within hours, along with the abundance of sperm and fertilization-related transcripts detected in the ovaries sample, corroborate previous observations [2] that fertilization occurs immediately following sperm ingestion and ovulation, adjacent to the ovaries within the gastrovascular cavity. Future molecular studies characterizing expression in sperm and eggs prior to and during fertilization will provide further insight into the dynamics of fertilization in cubozoans.

## Conclusions

Whereas most cubozoans are difficult to study in their natural settings, *Alatina alata* is becoming a useful model for evolutionary and molecular studies because mature adults can be found predictably in near-shore waters. In this study, we generated a new genomic resource for *A. alata*, a transcriptome of multiple adult tissues and larvae, and characterized patterns of expression of transcripts across several body parts of a female medusa and larval planulae. We identified a large suite of candidate genes implicated in predation and defense, vision and the phototransduction pathway, and sexual reproduction and embryogenesis. This new genomic resource and the candidate genes we have identified will be valuable for further investigating the evolution of distinctive features of cubozoans, and the evolution of cnidarians more broadly.

## Methods

### Specimen vouchers

Male and female *A. alata* medusae were collected in Bonaire, The Netherlands. All proper collection and export permits were obtained. Medusae were kept within a glass aquarium in filtered seawater for several hours. Males shed sperm into the water that was taken up by the female manubrium. Using light microscopy (1000x) to observe the ovaries, which are located within the gastrovascular cavity, confirmation was made of ovulation and sperm and egg interaction (i.e., putative fertilization) (Fig. 1c, d). No prey items were present within the stomach and associated gastric cirri or attached to the tentacle. A live female *A. alata* medusa undergoing internal fertilization was placed on ice, and using a sterile RNase-free disposable scalpel tissue samples were quickly excised from the gastric cirri, ovaries, tentacle, rhopalium (Fig. 1). A fifth sample consisting of thousands of swimming planulae that had developed from blastulae released from different females in the lab was also collected. All samples were placed in 2 ml cryovials and flash frozen with liquid nitrogen. Frozen samples were shipped via Cryoport to the Smithsonian Biorepository. Additionally, a single spawning *A. alata* female medusa was collected at Oil Slick Leap, Kralendijk on April 22, 2014, relaxed in 7.5 % Magnesium chloride, fixed and preserved in 8 % formalin, and deposited into the collection of the National Museum of Natural History, Washington, D.C. as a morphological voucher (USNM 1248604). No specific permissions were required from an ethics committee to conduct the research described herein as no humans or protected species were used.

### Sequencing

The five frozen tissue samples (gastric cirri, ovaries, tentacle (and adjoining pedalium base), rhopalium, and planulae) were sequenced at the University of Kansas Medical Center—Genomics Core (KUMC), where total RNA (0.5 ug) was used for library preparation for each sample. Illumina HiSeq 2500 Sequencing System was used to generate FASTQ files, which were de-multiplexed into individual sequences for further downstream analysis.

### Transcriptome assembly and post-assembly analyses

The 278 M paired end (100 bp) raw reads from five samples were analyzed on the Smithsonian Institution High Performance Cluster, SI/HPC, and filtered using the program TrimGalore! [121] with the adaptor trimming tool Cutadapt [122] and FastQC [123] (--quality 30 --phred33 --length 25) to remove Illumina lane and multiplex adaptors (overlapping by 1 bp). ALLPATHSLG error correction software [124] was used on the 265 M trimmed paired end reads (PAIRED_SEP option was set to 100), and unpaired reads following trimming to predict and correct sequencing errors (see [125]) and mitigate potential errors in transcriptome assemblies. All five samples (i.e., gastric cirri, ovaries, tentacle, rhopalium, and planulae) were pooled and assembled *de novo* into a reference transcriptome (FASTA format) for *A. alata* using Trinity (version trinityrnaseq_r20131110) [50, 51], with the following additional flags: --no_bowtie --normalize_reads--path_reinforcement_distance 75.

### Differential expression estimates and analyses

RNA-Seq by Expectation Maximization (RSEM) was run on each of the five samples separately to estimate transcript abundance (read counts). A single matrix was generated corresponding to expression values for all samples as normalized Trimmed Mean of M-values (TMM) [126]. EdgeR was then used to identify differentially expressed genes in the counts matrix (--dispersion 0.1) [55]; followed by differential expression analysis to extract all genes most significantly expressed, i.e., with *p*-values < =0.005 and with at least a fourfold change of differential expression (--matrix iso_r123456.TMM.fpkm.matrix -P 1e-3 -C 2). This EdgeR step generated a single expression matrix of the results of all pairwise comparisons between the five samples. Further, hierarchical clustering generated a heatmap indicating clustering of similarly expressed genes (vertical axis) plotted by sample type (horizontal axis), while maintaining column order by sample (--order_columns_by_samples). This was done for all differentially expressed transcripts for all five samples (Additional file 4); just for the medusa samples (Fig. 3a); and for each of the three subsets of differentially expressed candidate genes (Figs. 6a, 7a and 8a). Color-coding on the vertical access of each heatmap indicates gene clusters with similar mean expression levels. Gene cluster patterns were further subdivided into 10 K-mean subclusters, which were visualized as subcluster profile plots (Figs. 3b-k, 6, 7 and 8b-k). In the absence of biological replicates in this study, the specific significance of fold-change expression levels of each of the differentially expressed genes was of limited value, and we therefore chose to not further filter transcripts based on additional statistical analyses. Instead, all differentially expressed genes were targeted as candidates for narrowing our search for genes of interest by sample type. Furthermore, redoing the hierarchical clustering analysis on just the three subsets of candidate genes (putative venom, vision and sex genes) allowed us to hone in on gene clusters that were relevant to transcriptome functional annotation and profiling of *A. alata* samples types.

### Additional analyses

Venn diagrams were constructed using Venny [127]. Trinotate reports for each of the three sets of candidate genes investigated in this study (venom, vision and sex) were generated by filtering the original *A. alata* Trinotate report using this custom Python script: https://github.com/pbfrandsen/SI_scripts/blob/master/cheryl_trinotate.py.

### Gene tree reconstruction

Amino acid sequences corresponding to predicted ORFs (TransDecoder), or translated nucleotide sequences, from the *A. alata* transcriptome were aligned using MUSCLE (default parameters with 5 iterations) against other cnidarian homologs from NCBI Genbank for the respective candidate genes of interest. ProtTest v. 3.2 was used to determine the most appropriate model of amino acid evolution (i.e., LG + G or WAG + G + F, or BLOSS62 I-G-F) for each alignment. Shimodaira-Hasegawa-like branch support indices [128] and are shown at each node of the ML topology. All ORF alignments (.nex files) predicted from TransDecoder, and the corresponding gene tree reconstructions (.tre files) are available at: https://figshare.com/articles/Supplemental_Information_for_A_new_transcriptome_and_transcriptome_profiling_of_adult_and_larval_tissue_in_the_box_jellyfish_Alatina_alata_an_emerging_modelfor_studying_venom_vision_and_sex/3471425.

## Additional files

Additional file 1: Transcriptome statistics. Summary table of Trinity gene and transcript length distribution in whole transcriptome and filtered (fpkm = 1.5) transcriptome. Summary table of EdgeR and Trinotate results. (XLS 34 kb) Additional file 2: Trinotate report of candidate genes. A filtered Trinotate annotation report corresponding to annotations for all 651 candidate genes investigated for their putative role in venom, vision and sex (and early development) in the *A. alata* transcriptome. (XLS 3918 kb) Additional file 3: Trinotate report of Cnidaria genes. A filtered Trinotate annotation report corresponding to *A. alata* transcripts whose top BLASTX/BLASTP hits corresponded to cnidarian genes/ proteins; summary table and pie chart included. (XLS 11650 kb) Additional file 4: Heatmap and transcript DE matrix for five samples. Heatmap and corresponding matrix file used in hierarchical clustering (EdgeR) of ~10 K transcripts differentially expressed across *A. alata* medusa (gastric cirri, ovaries, tentacle (with pedalium base), rhopalium) and planulae samples (columns) (of the identified ~32 K Trinity transcripts). (XLS 1463 kb) Additional file 5: Heatmap and gene DE matrix for four medusa samples. Corresponds to Fig. 3a-k in the text. Matrix file corresponding to the heatmap showing hierarchical clustering (EdgeR) of the 2916 genes differentially expressed across *A. alata* medusa samples (gastric cirri, ovaries, tentacle (with pedalium base), rhopalium) (of the identified ~20 K Trinity genes). (MATRIX 107 kb) Additional file 6: Top 50 DE genes of medusa samples. Corresponds to Fig. 4b (Venn) in the text. Spreadsheet corresponding to the Venn diagram of the top fifty most highly expressed genes across the four medusa samples (from matrix in Additional file 5). Core genes are identified for each sample (gastric cirri, ovaries, tentacle (with pedalium base) and rhopalium). Detailed statistics (fpkm, counts, DE values for top 50 ranked genes by sample) summarized in histograms in Fig. 5a-d. Annotations are provided from the Trinotate report. (XLSX 66 kb) Additional file 7: Venom heatmap. Corresponds to Fig. 6a-k in the text. Matrix file corresponding to matrix used for hierarchical clustering to generate the heatmap (EdgeR) of the 450 genes putative genes implicated in venom in *A. alata* medusa (gastric cirri, ovaries, tentacle (with pedalium base) and rhopalium) and planulae samples. Annotations are provided from the Trinotate report. (XLSX 69 kb) Additional file 8: Vision heatmap. Corresponds to Fig. 7a-k in the text. Matrix file corresponding to matrix used for hierarchical clustering to generate the heatmap (EdgeR) of the 97 genes putative genes implicated in vision in *A. alata* medusa (gastric cirri, ovaries, tentacle (with pedalium base) and rhopalium) and planulae samples. Annotations are provided from the Trinotate report. (XLSX 27 kb) Additional file 9: Sex heatmap. Corresponds to Fig. 8a-k in the text. Matrix file corresponding to matrix used for hierarchical clustering to generate the heatmap (EdgeR) of the 104 genes putative genes implicated in sex in *A. alata* medusa (gastric cirri, ovaries, tentacle (with pedalium base) and rhopalium) and planulae samples. Annotations are provided from the Trinotate report. (XLSX 31 kb) Additional file 10: Top 50 DE genes of planulae sample. Spreadsheet corresponding to the top fifty most highly expressed transcripts in the planulae sample of those differentially expressed (DE) across all five *A. alata* samples (gastric cirri, ovaries, tentacle (with pedalium base), rhopalium, planulae) (from matrix in Additional file 4). Annotations are provided from the Trinotate report. (XLSX 19 kb) Additional file 11: J-Crystallins alignment. Figure corresponding to the amino acid sequence alignments of the three non-homologous cubozoan J-crystallins (J1, J2, J3). Proteins corresponding to *T. cystophora* J1, J2 and J3 crystallins and to *C. fleckeri* J3 crystallins from NCBI were aligned against the respective J1, J2 and J3 crystallin homolog for *A. alata* in this study. Boxes around residues indicate similarity in amino acid sequence, with black boxes corresponding to consensus regions. Sequences were aligned using MUSCLE (default parameters with 5 iterations). *A. alata* amino acid sequences correspond to predicted ORFs (TransDecoder), except for comp57165 which is a frame 1 translation of the Trinity transcript. (PNG 1047 kb)
