# Erratum to: A new transcriptome and transcriptome profiling of adult and larval tissue in the box jellyfish *Alatina alata*: an emerging model for studying venom, vision and sex

**DOI:** 10.1186/s12864-016-3305-y

**Published:** 2016-11-28

**Authors:** Cheryl Lewis Ames, Joseph F. Ryan, Alexandra E. Bely, Paulyn Cartwright, Allen G. Collins

**Affiliations:** 1 Department of Invertebrate Zoology, National Museum of Natural History, Smithsonian Institution, Washington, DC 20013 USA; 2 Biological Sciences Graduate Program, University of Maryland, College Park, MD 20742 USA; 3 Whitney Laboratory for Marine Bioscience, University of Florida, St Augustine, FL 32080 USA; 4 Department of Biology, University of Florida, Gainesville, FL 32611 USA; 5 Department of Biology, University of Maryland, College Park, MD USA; 6 Department of Ecology and Evolutionary Biology, University of Kansas, Lawrence, KS 66045 USA; 7 National Systematics Laboratory, NOAA Fisheries, National Museum of Natural History, Smithsonian Institution, Washington, DC USA; 8 Smithsonian Institution, National Museum of Natural History, 10th and Constitution Ave NW, Washington, DC 20560-0163 USA

## Erratum

After publication of our article [1], it was brought to our attention by a colleague that in describing the “7 transmembrane receptor (rhodopsin family)” G protein-coupled receptor (GPCR) transcripts of *Alatina alata* as “opsins” in Fig. 11 and the results and discussion sections of the paper, we misrepresented the data, as Opsins are just one subfamily of rhodopsin family GPCRs. We regret that this important detail was overlooked prior to publication, and we apologize for any inconvenience caused by this error.

**Fig. 11:**
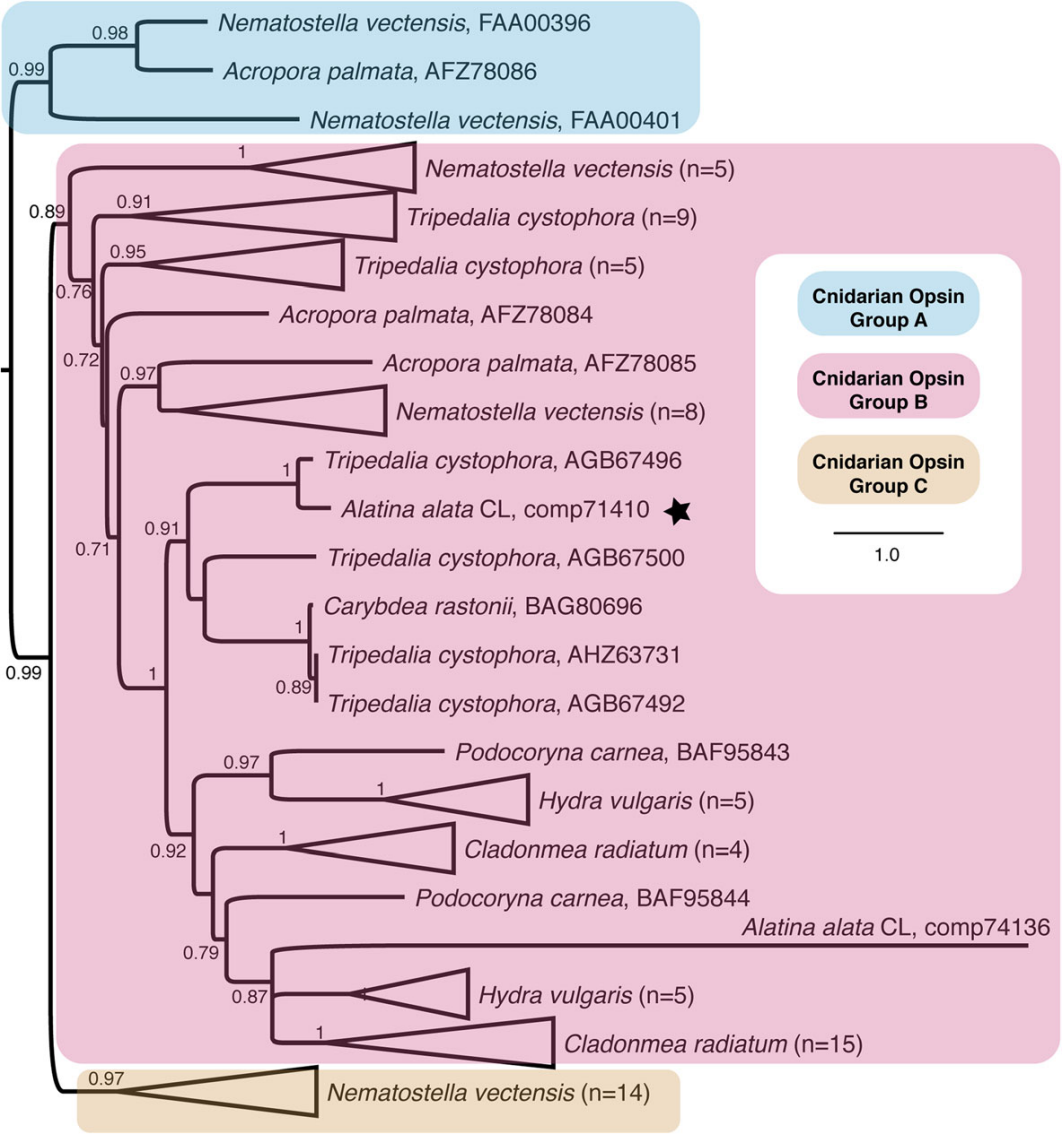
**b** Corrected cnidarian opsin gene tree. ML topology of known opsin homologs (*n =* 82) in cnidarian taxa from NCBI Genbank, and two opsin ORFs predicted from *A. alata* transcriptome components (comp71410, comp74136). Assumes the LG + I + G model of amino acid evolution, as specified as most appropriate by ProtTest v. 3.3. Shimodaira-Hasegawa-like branch support indices are shown at each node if greater than 0.5. The star indicates that in *A. alata*, the opsin transcript corresponding to comp71410 is expressed exclusively in the rhopalium, while comp74136 is most abundant in the rhopalium but also expressed in extraocular medusa samples and planulae

More stringent analysis of the *A. alata* “7 transmembrane receptor (rhodopsin family)” GPCRs revealed only two likely opsins (comp71410 and comp74136) based on the alignment of their conserved lysine (K), for retinal binding, with that of the bovine rhodopsin reference protein. Therefore, all mention of *A. alata* “opsin” expression in the original article should be interpreted as “rhodopsin family GPCR” expression, except when referencing the two putative opsins: comp71410 (exclusively expressed in the rhopalium) and comp74136 (most abundant in the rhopalium, but also expressed in extraocular medusa tissues and planulae). In light of these new findings, Fig. 11 “cnidarian opsin gene tree” and all related references in our original article should be disregarded. Figure 11 is replaced here with Fig. 11b, a corrected gene tree reconstructed using only cnidarian opsin proteins from NCBI Genbank and the two putative *A. alata* opsin ORFs. The gene tree reconstructions (.tre file) and corresponding alignment are available at: https://figshare.com/articles/Supplemental_Information_for_A_new_transcriptome_and_transcriptome_profiling_of_adult_and_larval_tissue_in_the_box_jellyfish_Alatina_alata_an_emerging_modelfor_studying_venom_vision_and_sex/3471425.

The discovery that *A. alata* opsin diversity is less broad than previously suggested does not impact the overall conclusions of our paper regarding opsin expression. Our updated findings still suggest that *A. alata* opsin is most abundant in the rhopalium, but also expressed in planulae, which have eyes spots, as well as in extra-ocular tissue types in the medusa, suggesting the presence of yet undescribed photoreceptors. Additional non-opsin rhodopsin family GPCRs (*n =* 31), whose specific identities await further analyses, are expressed in both the *A. alata* medusa samples and planulae. Previously we touched on the apparent diversity of *A. alata* rhodopsin family GPCRs, based on the fact that some of our BLAST hits for those sequences corresponded to non-opsin GPCRs (e.g., dopamine receptor, prostaglandin E2 receptor, melanocyte-stimulating hormone receptor), while others corresponded to various opsin types (e.g., lens eye opsin, peropsin, melanopsin).
