# Supplementary figures and images for: A new transcriptome and transcriptome profiling of adult and larval tissue in the box jellyfish Alatina alata: an emerging model for studying venom, vision and sex
Source: BMC Genomics. 2016 Aug 17;17:650. doi: 10.1186/s12864-016-2944-3 (PMC4989536; doi:10.1186/s12864-016-2944-3)

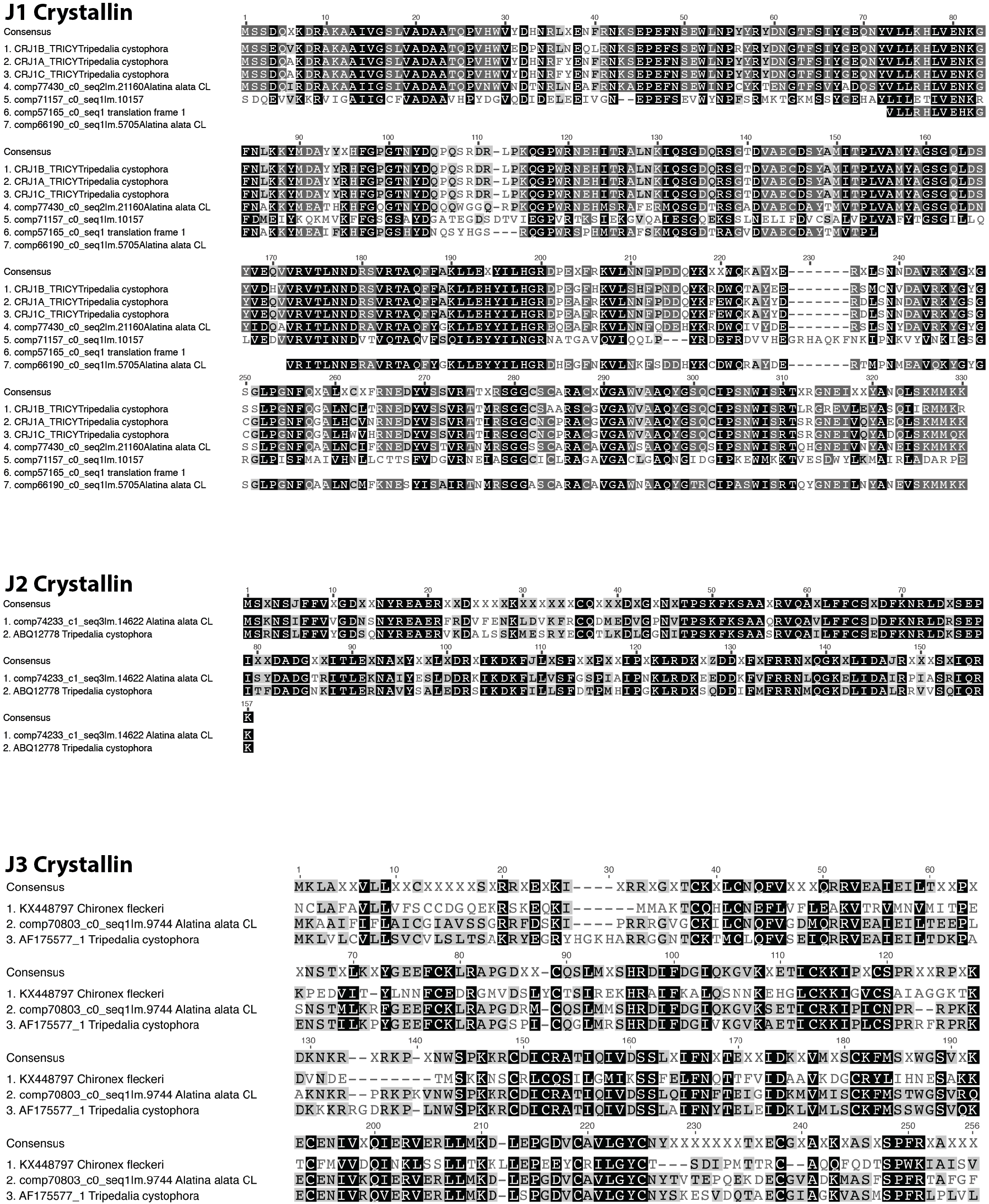

Supplement: Additional file 11: — J-Crystallins alignment. Figure corresponding to the amino acid sequence alignments of the three non-homologous cubozoan J-crystallins (J1, J2, J3). Proteins corresponding to T. cystophora J1, J2 and J3 crystallins and to C. fleckeri J3 crystallins from NCBI were aligned against the respective J1, J2 and J3 crystallin homolog for A. alata in this study. Boxes around residues indicate similarity in amino acid sequence, with black boxes corresponding to consensus regions. Sequences were aligned using MUSCLE (default parameters with 5 iterations). A. alata amino acid sequences correspond to predicted ORFs (TransDecoder), except for comp57165 which is a frame 1 translation of the Trinity transcript. (PNG 1047 kb) [file 12864_2016_2944_MOESM11_ESM.png]
